# Exploring the Feasibility and Usability of Smartphones for Monitoring Physical Activity in Orthopedic Patients: Prospective Observational Study

**DOI:** 10.2196/44442

**Published:** 2023-07-04

**Authors:** Arash Ghaffari, Rikke Emilie Kildahl Lauritsen, Michael Christensen, Trine Rolighed Thomsen, Harshit Mahapatra, Robert Heck, Søren Kold, Ole Rahbek

**Affiliations:** 1 Interdisciplinary Orthopaedics Aalborg University Hospital Aalborg Denmark; 2 Alexandra Institute Aarhus Denmark; 3 Danish Technological Institute Aarhus Denmark; 4 Department of Chemistry and Bioscience Aalborg Uuniversity Aalborg Denmark

**Keywords:** remote monitoring, physical activity, step count, smartphone application, wearable sensors, mixed effects modeling, step count prediction, mobile phone

## Abstract

**Background:**

Smartphones are often equipped with inertial sensors that measure individuals’ physical activity (PA). However, their role in remote monitoring of the patients’ PAs in telemedicine needs to be adequately explored.

**Objective:**

This study aimed to explore the correlation between a participant’s actual daily step counts and the daily step counts reported by their smartphone. In addition, we inquired about the usability of smartphones for collecting PA data.

**Methods:**

This prospective observational study was conducted among patients undergoing lower limb orthopedic surgery and a group of nonpatients as control. The data from the patients were collected from 2 weeks before surgery until 4 weeks after the surgery, whereas the data collection period for the nonpatients was 2 weeks. The participant’s daily step count was recorded by PA trackers worn 24/7. In addition, a smartphone app collected the number of daily steps registered by the participants’ smartphones. We compared the cross-correlation between the daily steps time series obtained from the smartphones and PA trackers in different groups of participants. We also used mixed modeling to estimate the total number of steps, using smartphone step counts and the characteristics of the patients as independent variables. The System Usability Scale was used to evaluate the participants’ experience with the smartphone app and the PA tracker.

**Results:**

Overall, 1067 days of data were collected from 21 patients (n=11, 52% female patients) and 10 nonpatients (n=6, 60% female patients). The median cross-correlation coefficient on the same day was 0.70 (IQR 0.53-0.83). The correlation in the nonpatient group was slightly higher than that in the patient group (median 0.74, IQR 0.60-0.90 and median 0.69, IQR 0.52-0.81, respectively). The likelihood ratio tests on the models fitted by mixed effects methods demonstrated that the smartphone step count was positively correlated with the PA tracker’s total number of steps (*χ*^2^_1_=34.7, *P*<.001). In addition, the median usability score for the smartphone app was 78 (IQR 73-88) compared with median 73 (IQR 68-80) for the PA tracker.

**Conclusions:**

Considering the ubiquity, convenience, and practicality of smartphones, the high correlation between the smartphones and the total daily step count time series highlights the potential usefulness of smartphones in detecting changes in the number of steps in remote monitoring of a patient’s PA.

## Introduction

### Background

Daily physical activity (PA) is crucial for maintaining physical, mental, and social health [1]. For patients undergoing orthopedic surgery, resuming PA as soon as possible is vital to enhance recovery and prevent complications [2]. In addition, assessing PA after surgery can provide valuable information regarding a patient’s health condition, allowing for individualized rehabilitation based on the patient’s condition and demands [3-5]. However, some limitations and challenges exist regarding the measurement of PA. Current patient-reported outcome measures (PROMs) such as questionnaires and surveys might seem convenient for evaluating the level of PA; however, they have limitations such as low patient adherence, floor effects, and recall bias and are inefficient in measuring walking as an important PA [6]. In addition, PROMs are often obtained at specific and broad intervals. Therefore, the objective measurement of PA after discharge is of increasing interest [7].

Smartphones and other digital devices are currently equipped with sensors allowing the quantification of an object’s motion by converting inertial forces into measurable electrical signals [8]. This makes them valuable tools for remotely monitoring patients’ PA during recovery after surgery. Smartphones have also become increasingly prevalent across all age groups and are now ubiquitous [9]. For instance, in Denmark, 90% of the population has access to smartphones [10], making them a widespread technology with the potential for broad societal impact. Using smartphones in remote monitoring the patients also offers the possibility of applying supplementary PROMs. A recent study on patients undergoing hip replacement surgeries demonstrated the patients’ interest in using smartphone apps and learning how to use wearable sensors [11]. Collecting activity data and PROMs with a smartphone for this group of patients has proven feasible [12].

Given the increasing prevalence of smartphones in the general population and their growing application in telemedical methods, these devices can play a prominent role in collecting objective PA data. However, their capability has not been fully explored, especially in free-living settings and over extended periods, such as follow-up after surgeries. In addition, some uncertainties have been discussed regarding the validity of the measurements, as the patients usually do not carry their smartphones all the time [13]. Specifically, changing daily life routines during and immediately after surgery may cause the patients not to carry their devices as usual. Accordingly, the amount and the significance of the nonmeasured activity in the perioperative periods are unknown.

### Objectives

In this study, we explored the utility of smartphones in measuring daily PA compared with wearable sensors in orthopedic patients during the perioperative period. The PA trackers were used to record step counts during regular continuous walking, sporadic walking, and slow continuous walking. The primary objective of this study was to determine the correlation between the daily step counts obtained from smartphones and the step counts registered by the PA trackers during these different types of walking. In addition, we investigated the ability of smartphones to predict the total number of daily steps taken during each type of walking. The secondary objective was to evaluate the usability of a smartphone app designed to collect health data.

## Methods

### Study Design and Setting

This prospective observational study was conducted at the Aalborg University Hospital, Denmark, between November 2021 and August 2022. The project was registered at North Jutland Research Database in Denmark (2021-119).

### Ethics Approval

This study was approved by the Regional Committee on Health Research Ethics (reference 2021-000438). This study complies with the Strengthening the Reporting of Observational Studies in Epidemiology guidelines [14].

### Participants

#### Overview

We included 2 groups of participants in this study to compare the results of the patients undergoing orthopedic surgeries with those of a control group. First, all participants were informed about the study process and were asked to sign informed consent forms. Subsequently, the participants were instructed to install and use the smartphone app and the PA trackers and transfer the data.

#### Patients

Patients undergoing lower limb orthopedic surgery were eligible for inclusion if they were smartphone users. No limitation was placed regarding the participant’s age or the type of surgery. However, older, frail patients who required a wheelchair for ambulation or who could not walk independently were not included.

Patients’ data were collected from at least 2 weeks before surgery until 4 weeks after surgery.

#### Nonpatients

We also included volunteers without orthopedic problems as the control group. Data regarding the step counts for at least 14 consecutive days were collected in this group.

### Data Sources and Measurements

#### Participants’ Characteristics

The patients’ basic and demographic information, including their age, sex, BMI, comorbidities (history of medical illness), and previous orthopedic surgery on the lower limbs, were registered in a REDCap (Research Electronic Data Capture; Vanderbilt University) database hosted by the North Jutland Region, Denmark [15].

#### PA Tracker

**Figure 1:**
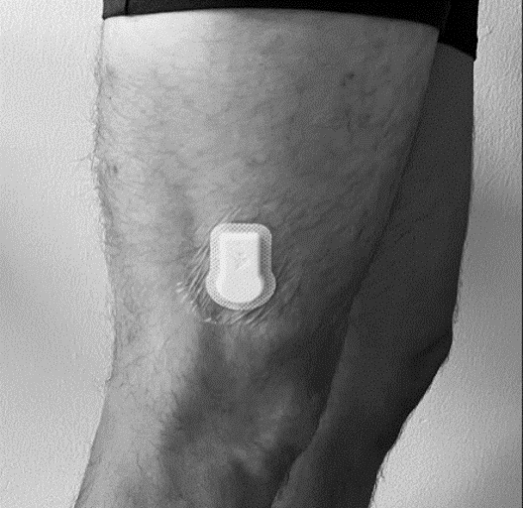
The photograph demonstrates the SENS Motion physical activity tracker at the lateral side of the distal thigh of 1 of the participants in the study.

SENS sensors (SENS Motion) were used to record the patients’ daily number of steps. SENS Motion is a wearable PA sensor worn as a patch on the lateral distal thigh and collects PA data by registering 3D linear acceleration data (Figure 1). Some studies have investigated the reliability and validity of the SENS PA trackers’ measurements [16,17] and demonstrated favorable results. As the sensors were attached 24/7 to the patients, we considered their measurements as the total daily step counts. To ensure that the patients wore the sensors for the entire duration, we observed the sensors’ relative temperature data in addition to the linear acceleration daily time series.

The SENS Motion algorithm calculates the number of steps taken during sporadic and continuous walking, as well as training, in three different categories:

1. *Steps-1*: Summarized number of steps during continuous walking and training, based on analysis in the frequency domain.
2. *Steps-2*: Steps taken during sporadic and irregular walking where no continuous frequency can be recognized in the 5-second interval are summarized as 2 steps per 5-second interval.
3. *Steps-3*: Steps taken during slow walking where a continuous frequency can be recognized, but the intensity of the accelerations is lower than that in usual walking.

We calculated the *total PA tracker steps* as the sum of the 3 abovementioned variables.

#### Smartphone App (OrtoApp)

OrtoApp (Alexandra Institute) is a smartphone app developed to collect step counts and PA data from the Apple HealthKit application programming interface (API) on iOS and the Google Fit API on Android smartphones [18,19]. During the study, the app was installed on patients’ smartphones and automatically recorded the steps registered by the Apple HealthKit and the Google Fit APIs. Furthermore, if a person also wears a smartwatch, the Apple HealthKit and Google Fit APIs will collect the data from both devices (the smartwatch and the smartphone), and the step counts will be calculated based on both inputs.

In addition, OrtoApp allows users to record their daily mood and pain levels on an 11-point visual analog scale (0-10). However, we did not use the data regarding the pain and mood scores in this study.

#### Usability of the Smartphone App and PA Tracker

We used the System Usability Scale (SUS) to evaluate participants’ experience with the smartphone app and the PA tracker. The SUS is developed as a survey scale that allows quick and easy assessment of the usability of a given product or service [20]. The original SUS instrument comprises 10 statements scored on a 5-point scale of the strength of agreement. Final SUS scores can range from 0 to 100, with higher scores indicating better usability [21]. In this study, we used the translated and validated Danish version of the SUS [22].

After the data collection period was over, we assessed the usability only in the patient group by distributing the SUS questionnaire via the REDCap web application.

### Steps Data Analysis

We generated 5 time series for each participant, including 1 for the daily steps recorded by the smartphones and 4 for the daily PA trackers’ measurements (steps-1, steps-2, steps-3, and PA tracker total steps). These time series were then plotted for each participant, and we compared the smartphone data’s time series with the different variables of the PA trackers using cross-correlation. Before conducting the cross-correlation analysis, we differentiated the time series data to remove any trends or changes in the mean that may have affected the results. This was done by calculating the difference between consecutive time points (days). Next, we calculated the cross-correlation between the resulting time series using a standard method [23]. We specifically calculated the cross-correlation at 0 days lag (ie, the same day) to assess the immediate relationship between the variables. We used Fisher Z transformation to calculate the 95% CI for the correlation coefficients and to compare the correlation coefficients [24]. The comparisons were performed between various groups based on different criteria, including patient or nonpatient status, preoperative or postoperative status (for patients), age (>60 years or <60 years), comorbidities, history of lower limb surgery, day of data collection (weekday—Monday through Friday—or weekend—Saturday and Sunday), content type of the smartphone used, and the use of a smartwatch.

In addition, we applied mixed effects models to investigate whether the smartphone’s step counts could predict the total number of steps. Only the data from the patient group were used for mixed effects modeling. To prepare the data for regression analysis, we applied the moving average method to calculate the average values for the 3 preceding days (trailing moving average with a window of 3 days). In time series data analysis, the moving average method helps discover certain traits by smoothing the variations and reducing the noise [23]. Subsequently, we scaled the data to have a mean 0 and a SD equal to 1.

We used different subjects as random intercepts in the models and by-subject PA tracker–smartphone steps slope variance as random slopes. We included the following variables and all possible interaction effects between the variables to fit the models:

1. Smartphone steps:
  Scaled 3-days moving average as a continuous variable
2. Participants’ characteristics:
  Age in years as a continuous variable
  Sex as a categorical variable (male or female)
  BMI in kg/m^2^ as a continuous variable
  Comorbidity as a categorical variable (yes or no)
  History of lower limb surgery as a categorical variable (yes or no)
3. Characteristics of data collection day:
  Preoperative versus postoperative as a categorical variable
4. Smartphone health app:
  Apple HealthKit versus Google Fit as a categorical variable
5. Smartwatch:
  Using a smartwatch as a categorical variable (yes or no).

The variables included in the best-performing models were selected by backward elimination, that is, if they did not improve the model, the variables were omitted.

Four models were created for the different variables from the PA tracker (steps-1, steps-2, steps-3, and PA tracker total steps). In the best-fitted models for the steps-2, steps-3, and PA tracker total steps, the selected variables were the period (preoperative or postoperative) and the presence of comorbidities in addition to smartphone steps. However, in the PA steps-1 model, the history of medical disease did not improve the model performance and hence was excluded.

The coefficients for the fixed and random effects variables in the best-fitted models and the performance metrics for the goodness of fit for the models (described in *Statistical Methods* section) were computed. The 95% prediction intervals for the models were created and plotted by bootstrapping techniques.

### Statistical Methods

We used the R statistical package (version 4.1.0; R Foundation for Statistical Computing) for the statistical analyses and *lme4* package [25] for the mixed effects models.

Descriptive statistics were used to describe participants’ basic information. The counts and percentages were used for the discrete variables, including the number and sex of the participants and the number of days for data collection. Means and SDs were used to describe the participants’ age and BMI. We presented the cross-correlation coefficients between the time series as means and 95% CIs. The SUS values for the smartphone app and PA trackers were provided as median and IQR.

Mixed effects models were created using the restricted maximum likelihood approach. The repeated measures and covariance matrix were modeled as unstructured. No violation of the model assumptions regarding the linearity, homoscedasticity, and normality of residuals was detected. The goodness of fit of the models was assessed by calculating the deviance, Akaike information criterion, Bayesian information criterion [26], intraclass correlation coefficient, and conditional and marginal pseudo-*R*^2^ [27]. Marginal pseudo-*R*^2^ represents the variance explained by the fixed effects, whereas conditional pseudo-*R*^2^ is interpreted as a variance explained by the entire model, that is, both fixed and random effects. The scaled step counts were back transformed into actual values in the plots. We compared the best-fitted models with and without the smartphone step counts by using likelihood ratio tests to calculate *P* values. The significance level was set at α=.05.

## Results

### Participants’ Characteristics

Overall, 35 participants were included in the study; however, 4 participants were excluded, and data of 31 participants (n=21, 68% patients and n=10, 32% nonpatients) were analyzed. Table 1 presents the characteristics of the participants.

**Table 1:** Characteristics of the participants in the study.

Variable			Patient (n=21)		Nonpatient (n=10)		Total (n=31)
Age (years), mean (SD)			57.6 (16.4)		49.9 (10.2)		55.1 (14.9)
Sex (female), n (%)			11 (52)		6 (60)		17 (55)
History of lower limb surgery, n (%)			16 (76)		1 (10)		17 (55)
Comorbidities, n (%)			12 (57)		5 (50)		17 (55)
BMI (kg/m^2^), mean (SD)			28.7 (5.2)		26.9 (5.3)		28.1 (5.3)
**Smartphone health app, n (%)**							
	Google Fit	17 (81)		8 (80)		25 (81)	
	Apple HealthKit	4 (19)		2 (20)		6 (19)	
Smartwatch, n (%)			4 (19)		2 (20)		6 (19)

Participants were excluded owing to surgery cancellation (2/4, 50%) and technical problems with the sensor (1/4, 25%) or the smartphone app (1/4, 25%). In addition, data from 3 patients only contained preoperative data because one of the patients discontinued collecting data after the surgery, the surgery was postponed in another patient, and the sensor was lost in the operating room in the third patient. The time series from patients who only had preoperative data were used for cross-correlation analysis and comparison, but they were not included in the regression analysis.

In the patient group, the surgical procedures performed included total hip arthroplasty (11/21, 52%), total knee arthroplasty (5/21, 24%), osteosynthesis (3/21, 14%), and high tibial osteotomy (2/21, 10%). The most common symptoms were pain (20/21, 95%), walking problems (18/21, 86%), and joint stiffness (7/21, 33%). In total, 17 participants had comorbidities, and 15 participants took daily medications for high blood pressure (8/21, 38%), heart disease (3/21, 14%), diabetes (2/21, 10%), high cholesterol (2/21, 10%), and other diseases (4/21, 19%). Regarding the history of lower limb surgeries, 7 patients had previous knee surgery, 3 had hip surgery, and 6 had other surgeries. In the nonpatient group, 1 person had previous knee surgery.

We collected 1067 days of data (915 days from the patients and 152 days from the nonpatients). The number of data collection days per patient was between 10 and 16 (mean 14) days in the nonpatient group and between 39 and 69 (mean 49) days in the patient group, except for 3 patients with only preoperative data (with 8-, 10-, and 13-day data).

### Step Count Analysis

The median and IQR for the step counts from the PA tracker and the smartphone and the percentages of different step types (steps-1, steps-2, and steps-3) in the total PA tracker step counts in various groups of the participants are provided in Table 2.

**Table 2:** Median and IQR of step counts measured by smartphone and physical activity (PA) tracker by participant characteristics and distribution of step types (steps-1, steps-2, and steps-3) within PA tracker total steps.

Variables		Days, n	Smartphone steps, median (IQR)	PA tracker total steps, median (IQR)	PA tracker total steps composition, median (IQR)		
					Steps-1^a^	Steps-2^a^	Steps-3^a^
**Group**							
	Patient	915	2000 (700-4800)	6500 (3800-10,800)	48 (35-57)	21 (16-33)	29 (24-32)
	Nonpatient	152	4600 (2300-9400)	14,800 (10,600-18,300)	58 (52-65)	17 (14-19)	25 (20-28)
**Period**							
	Preoperative	394	2700 (1000-6300)	9600 (6000-13,600)	53 (47-60)	18 (14-23)	28 (24-31)
	Postoperative	521	1400 (400-35,000)	5300 (2800-7700)	42 (22-51)	27 (18-44)	30 (24-33)
**Age (years)**							
	≤60	560	3200 (900-6500)	8000 (4400-13,500)	54 (45-61)	18 (14-24)	27 (23-31)
	>60	507	1600 (600-3600)	6800 (4100-11,000)	44 (27-54)	24 (17-39)	29 (23-33)
**Sex**							
	Female	540	2600 (700-6400)	9300 (5000-14,500)	49 (36-58)	20 (16-34)	27 (22-31)
	Male	527	2000 (800-4500)	6200 (4000-10,000)	50 (40-59)	19 (14-28)	30 (25-33)
**Comorbidity**							
	Negative	504	2100 (600-5300)	9500 (5200-14,300)	50 (40-59)	20 (16-30)	27 (22-31)
	Positive	563	2400 (900-5900)	6200 (3900-10,000)	49 (37-58)	20 (15-30)	29 (25-33)
**Previous surgery**							
	Negative	394	3100 (1200-7300)	7800 (4900-13,800)	55 (47-61)	17 (14-22)	28 (24-32)
	Positive	673	1900 (600-4500)	7100 (3900-11,500)	46 (30-55)	22 (17-38)	29 (23-32)
**Day of week**							
	Weekday	755	2200 (800-5600)	7200 (4200-12,000)	50 (38-59)	20 (15-30)	28 (23-32)
	Weekend	312	2200 (700-5100)	7900 (4300-12,800)	50 (38-58)	20 (16-29)	28 (24-32)
**Smartwatch**							
	Yes	217	2900 (1500-5900)	6800 (4500-11,100)	42 (27-55)	28 (19-40)	29 (25-32)
	No	850	2000 (600-5300)	7600 4200-12,600)	51 (42-59)	19 (15-27)	28 (23-32)
**Smartphone health app**							
	Apple HealthKit	904	2200 (800-5600)	7300 (94,100-12,200)	49 (38-58)	20 (16-29)	29 (24-32)
	Google Fit	163	2400 (800-4800)	8400 (94,800-12,400)	54 (39-60)	18 (13-31)	27 (23-31)
All participants		1067	2200 (800-5500)	7400 (4300-12,400)	50 (38-59)	20 (15-30)	28 (23-32)

^a^Correspond to the proportions of total PA tracker steps in percentages.

**Figure 2:**
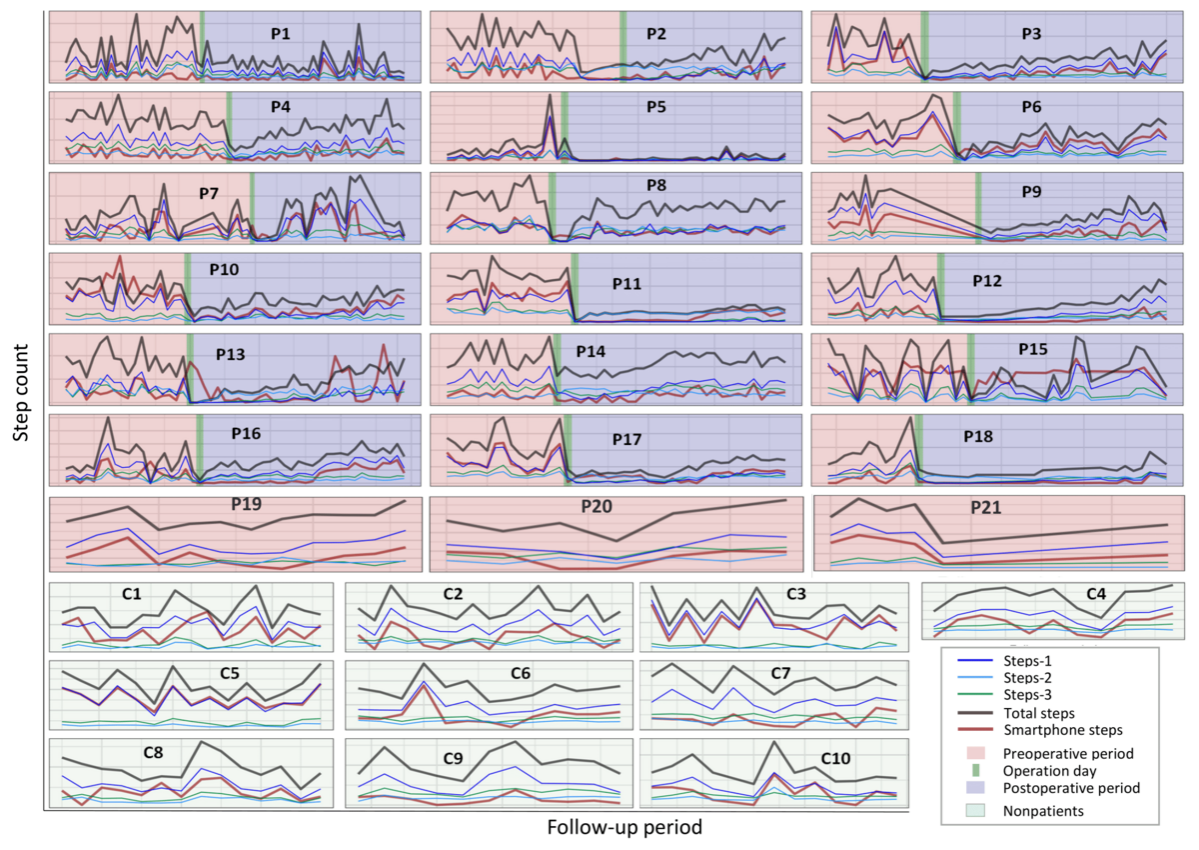
The upper panel shows the time series for step counts recorded by the smartphone and physical activity tracker for each patient (P) before and after the surgery, whereas the lower panel displays the same for nonpatient participants (C). Each plot corresponds to 1 participant, and the bold black font indicates their ID, which matches the IDs in Table 3. In the patient group, each gray horizontal gridline represents 5000 steps, and each gray vertical gridline represents 5 days. In the nonpatient group, each gray horizontal gridline represents 5000 steps, and each gray vertical gridline represents 2 days.

In Figure 2, the time series data for each patient during the preoperative and postoperative periods and for the nonpatient group are presented for both the smartphone and PA trackers. Table 3 shows the cross-correlation coefficients (*r*) at lag 0 between the smartphone time series and the time series for different PA tracker step counts (steps-1, steps-2, steps-3, and total steps) for each participant in the study.

Table 4 displays the median and IQR of the cross-correlation coefficients between the daily step count time series of smartphones and PA trackers for various variables (steps-1, steps-2, steps-3, and total steps).

**Table 3:** Cross-correlation at lag 0 between smartphone and physical activity (PA) tracker step count variables, with correlation coefficients (r) and *P* values for each participant^a^.

ID	Group	Steps-1 vs smartphone steps		Steps-2 vs smartphone steps		Steps-3 vs smartphone steps		PA tracker total steps vs smartphone steps	
		*r*	*P* value	*r*	*P* value	*r*	*P* value	*r*	*P* value
P1	Patient	0.70	<.001	0.40	.001	0.47	<.001	0.60	<.001
P2	Patient	0.66	<.001	0.19	.20	0.62	<.001	0.65	<.001
P3	Patient	0.92	<.001	0.26	.009	0.41	<.001	0.84	<.001
P4	Patient	0.78	<.001	0.57	<.001	0.72	<.001	0.73	<.001
P5	Patient	0.97	<.001	0.77	<.001	0.78	<.001	0.93	<.001
P6	Patient	0.94	<.001	0.45	.003	0.74	<.001	0.91	<.001
P7	Patient	0.72	<.001	0.48	<.001	0.57	<.001	0.68	<.001
P8	Patient	0.88	<.001	0.48	.001	0.63	<.001	0.75	<.001
P9	Patient	0.86	<.001	0.44	.005	0.72	<.001	0.84	<.001
P10	Patient	0.78	<.001	0.34	.01	0.27	.052	0.67	<.001
P11	Patient	0.95	<.001	0.32	.03	0.93	<.001	0.96	<.001
P12	Patient	0.78	<.001	0.63	<.001	0.79	<.001	0.80	<.001
P13	Patient	0.30	.03	0.13	.40	0.11	.40	0.16	.30
P14	Patient	0.74	<.001	0.28	.06	0.66	<.001	0.74	<.001
P15	Patient	0.55	<.001	0.50	.002	0.51	.001	0.55	<.001
P16	Patient	0.82	<.001	0.27	.047	0.26	.07	0.70	<.001
P17	Patient	0.96	<.001	0.78	<.001	0.89	<.001	0.96	<.001
P18	Patient	0.92	<.001	0.64	<.001	0.82	<.001	0.90	<.001
P19	Patient (only preoperative)	0.92	.01	0.44	.001	0.30	.08	0.72	.10
P20	Patient (only preoperative)	0.83	.08	0.20	.02	0.46	.02	0.66	.30
P21	Patient (only preoperative)	0.73	.02	0.49	.02	0.45	.06	0.53	.08
C1	Nonpatient	0.66	.005	0.29	.20	0.26	.30	0.61	.01
C2	Nonpatient	0.90	<.001	0.15	.50	0.17	.50	0.82	<.001
C3	Nonpatient	0.84	.01	0.06	.80	0.00	.90	0.68	.01
C4	Nonpatient	0.91	.002	0.69	.02	0.88	.002	0.90	.002
C5	Nonpatient	0.78	.007	0.15	.50	0.11	.60	0.63	.007
C6	Nonpatient	0.78	.01	0.26	.30	0.39	.10	0.66	.01
C7	Nonpatient	0.86	.003	0.76	.006	0.51	.007	0.82	.003
C8	Nonpatient	0.99	<.001	0.52	.04	0.62	.01	0.96	<.001
C9	Nonpatient	0.55	.10	0.09	.70	0.24	.40	0.38	.10
C10	Nonpatient	0.82	.01	0.24	.40	0.54	.06	0.73	.01

^a^The corresponding time series are demonstrated in Figure 2.

**Table 4:** Cross-correlation coefficients and corresponding 95% CIs between daily smartphone step counts and different step counts from the physical activity (PA) tracker (steps-1, steps-2, steps-3, and total steps)^a^.

		Steps-1 vs smartphone steps, mean (95% CI)	*P* value		Steps-2 vs smartphone steps, mean (95% CI)		*P* value		Steps-3 vs smartphone steps, mean (95% CI)		*P* value		PA tracker total steps vs smartphone steps, mean (95% CI)		*P* value	
**Group**				.02				.80				.50				.07
	Patient	0.79 (0.72-0.85)			0.35 (0.23-0.47)				0.48 (0.38-0.58)				0.70 (0.62-0.77)			
	Nonpatient	0.88 (0.74-0.94)			0.33 (0.09-0.53)				0.41 (0.20-0.59)				0.77 (0.66-0.88)			
**Period**				.02				.06				.06				.02
	Preoperative	0.82 (0.72-0.89)			0.41 (0.21-0.59)				0.53 (0.37-0.67)				0.74 (0.62-0.83)			
	Postoperative	0.75 (0.62-0.84)			0.28 (0.16-0.39)				0.42 (0.31-0.52)				0.64 (0.53-0.74)			
**Age (years)**				.09				.10				.10				.10
	≤60	0.83 (0.73-0.89)			0.39 (0.27-0.53)				0.46 (0.34-0.56)				0.75 (0.65-0.82)			
	>60	0.79 (0.70-0.86)			0.30 (0.15-0.44)				0.48 (0.33-0.61)				0.70 (0.58-0.79)			
**Sex**				.40				.60				.30				.40
	Female	0.80 (0.69-0.88)			0.34 (0.18-0.48)				0.45 (0.30-0.57)				0.71 (0.58-0.81)			
	Male	0.82 (0.75-0.87)			0.36 (0.21-0.50)				0.50 (0.39-0.59)				0.74 (0.67-0.79)			
**Smartwatch**				.30				.60				.40				.97
	Yes	0.84 (0.61-0.94)			0.31 (0.01-0.55)				0.40 (0.15-0.60)				0.73 (0.46-0.87)			
	No	0.80 (0.74-0.85)			0.36 (0.24-0.47)				0.49 (0.39-0.58)				0.72 (0.65-0.78)			
**Smartphone health app**				.003				.40				.30				.01
	Apple HealthKit	0.84 (0.77-0.90)			0.37 (0.24-0.48)				0.49 (0.39-0.58)				0.75 (0.68-0.81)			
	Google Fit	0.63 (0.49-0.73)			0.25 (0.06-0.42)				0.35 (0.26-0.44)				0.53 (0.42-0.62)			
All participants		0.82 (0.64-0.90)	—^b^		0.30 (0.10-0.56)		—		0.45 (0.23-0.66)		—		0.70 (0.53-0.83)		—	

^a^*P* values for group comparisons are provided.

^b^Not available.

### Regression Models for Step Counts

Tables 5-7 present the coefficients for the fixed and random effects for the regression models that best fit the data for steps-1, steps-2, steps-3, and the total steps recorded by the PA trackers, along with the goodness-of-fit metrics.

**Table 5:** Fixed effects for the best-fitted models estimating daily step counts using smartphone step counts^a^.

Variables^a^		Models							
		Steps-1	*P* value	Steps-2	*P* value	Steps-3	*P* value	PA tracker total steps	*P* value
Intercept, α (95% CI)		0.33 (0.07 to 0.59)	<.001	0.68 (0.14 to 1.23)	.01	0.76 (0.32 to 1.2)	.002	0.67 (0.37 to 0.96)	<.001
**Slope, β (95% CI)**									
	Smartphone steps	0.82 (0.66 to 0.99)	<.001	0.34 (0.15 to 0.52)	.001	0.85 (0.56 to 1.14)	<.0001	0.85 (0.67 to 1.04)	<.001
	Period (postoperative)	−0.44 (−0.61 to −0.26)	<.001	−0.30 (−0.53 to −0.06)	.02	−0.52 (−0.72 to −0.32)	<.001	−0.47 (−0.65 to −0.30)	<.001
	Positive medical history	—^b^	—	−0.84 (−1.55 to −0.13)	.02	−0.53 (−1.09 to 0.03)	.06	−0.53 (−0.89 to −0.18)	.007

^a^The values in this table regard the scaled step counts.

^b^Not available.

**Table 6:** Random effects variances for the best-fitted models estimating daily step counts using smartphone step counts.

Random effects	Models			
	Steps-1	Steps-2	Steps-3	PA tracker total steps
The variance between individuals’ intercepts	0.22	0.47	0.32	0.15
The variance of PA tracker—smartphone steps slope between individuals	0.08	0.02	0.25	0.03
The variance of the residuals	0.07	0.20	0.18	0.10

**Table 7:** Goodness-of-fit metrics for the best-fitted models estimating daily step counts using smartphone step counts.

Model metrics	Models			
	Steps-1	Steps-2	Steps-3	PA tracker total steps
AIC^a^	402	1211	1111	663
BIC^b^	449	1263	1163	714
Deviance	38	1189	1089	641
ICC^c^	0.83	0.78	0.80	0.75
Conditional pseudo-*R*^2^	0.94	0.83	0.90	0.92
Marginal pseudo-*R*^2^	0.65	0.25	0.51	0.68

^a^AIC: Akaike information criterion.

^b^BIC: Bayesian information criterion.

^c^ICC: intraclass correlation coefficient.

**Figure 3:**
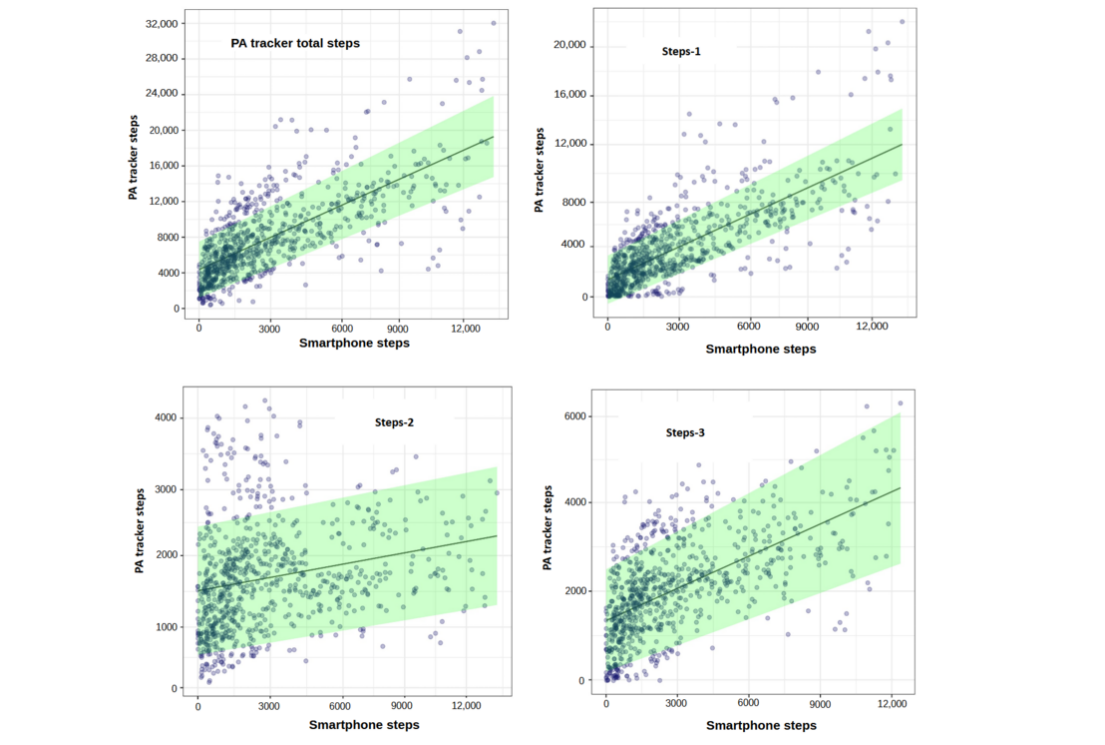
Results of the different models for estimating the daily step counts of the physical activity (PA) tracker, including the total steps, steps-1, steps-2, and steps-3. The mean values are depicted by solid lines, whereas the 95% prediction intervals are shown as light green shaded areas for each model.

Figure 3 displays the outcomes of various models, along with the 95% prediction intervals for all patients.

The models with the smartphone steps provided a better fit for the total step counts than the models without this variable. The likelihood ratio tests for comparing the selected models with and without smartphone steps demonstrated that the smartphone steps were positively correlated with PA tracker total steps (*χ*^2^_1_=34.7, *P*<.001), steps-1 (*χ*^2^_1_=36.8, *P*<.001), steps-2 (*χ*^2^_1_=11.4, *P*<.001), and steps-3 (*χ*^2^_1_=22.1, *P*<.001).

### Questionnaires and SUS Scores

Overall, 94% (17/18) of the patients filled out the questionnaires regarding SUS. The median scores were 78 (IQR 73-88) for the smartphone app and 73 (IQR 68-80) for the PA tracker, respectively. The scores were higher in female patients and in those aged <60 years (Table 8).

**Table 8:** The median and IQR of the System Usability Scale (SUS) scores for the smartphone app and the physical activity (PA) tracker for different age and sex groups.

Variables			SUS, median (IQR)		
			Smartphone app		PA tracker
**Age (years)**					
	≤60	93 (83-96)		88 (75-95)	
	>60	73 (65-76)		70 (66-73)	
**Sex**					
	Male	73 (65-80)		69 (65-76)	
	Female	83 (73-95)		78 (70-80)	
Total			78 (73-88)		73 (68-80)

## Discussion

### Principal Findings

In this study, we explored the feasibility of using smartphones for remote monitoring of orthopedic patients’ PA. To achieve this, we analyzed the correlation between the step counts recorded by a smartphone and a 24/7 PA tracker. Our results indicated a high correlation (*r*=0.70) between the time series of daily smartphone steps and daily PA tracker total steps. In addition, we found that the number of steps recorded by the smartphone was a strong predictor of changes in total daily steps. However, the absolute number of daily steps predicted using smartphone data was neither precise nor reliable.

The role of smartphones in remote monitoring of patients’ PA has not yet been clearly defined because of 2 main reasons. First, concerns persist regarding the validity and reliability of PA data collected by smartphones, as conflicting results have been reported in the literature [28]. For example, in a systematic review, the difference between smartphone measurements and a gold standard in a laboratory setting varied from 0.1% to 79.3%, and the reliability of smartphone measurements ranged from poor to excellent (intraclass correlation coefficient between 0.02 and 0.99) [13]. Second, the relationship between smartphone PA data and total PA data in different individuals is not fully understood and depends on various factors. The smartphone only records a variable proportion of the total daily PA, which is the time the person carries the device. Ignoring this point can lead to conflicting results, especially in studies with free-living settings. In this study, we investigated the relationship between the 2 variables and found that, despite considerable variability, a high correlation exists between smartphone step counts and total daily step counts.

The correlation between smartphone and total daily steps can vary significantly in a free-living setting, both between and within individuals. Several studies have found inferior results regarding the validity and reliability of smartphone measurements in free-living measurements compared with laboratory settings [29-31]. The variations may be even higher in orthopedic patients owing to pain and mobility issues during the early postoperative period, which could affect smartphone use and measurements. In a recent pilot study, Vorrink et al [32] found a mean correlation of 0.88 between smartphone and PA tracker measurements in a group of nonorthopedic patients, which was higher than the correlation we found in this study. However, we calculated the correlation between the time series after differentiating and detrending. Our analysis of different step count variables from the PA tracker revealed that the correlation with smartphone steps was the highest for steps-1 and the lowest for steps-2. We also found that the correlation between PA tracker’s steps-1 and PA data collected by smartphone was higher in the nonpatient group than in the patient group and during the preoperative period compared with the postoperative period. However, the correlation remained relatively high even during the postoperative period (*r*=0.64 and *r*=0.75 for total steps and steps-1, respectively). This discrepancy in the correlation could be attributed to the possibility that patients do not carry their smartphones as frequently during the postoperative period as they would under normal circumstances, or it could be because of the lower measurement accuracy in lower walking velocities, which has been demonstrated in previous studies [33,34]. Regarding the PA tracker’s steps-2 and steps-3, we could not find a significant difference in the correlations between subject groups with different characteristics (the *P* values were between .06 and .80).

Most participants (>80%) in our study used iOS smartphones, and we observed a stronger correlation in PA tracker’s steps-1 and total steps with smartphones equipped with Apple HealthKit APIs. However, we were unable to compare different smartphone types owing to the small sample size of participants with Google Fit API in our study. Several studies have investigated the impact of smartphone type on the accuracy and precision of PA measurements [35-38]. For instance, Höchsmann et al [38] found lower accuracy in an Android smartphone during low-velocity gait when compared with other smartphones and PA trackers. Moreover, we did not observe a high correlation between smartwatch users and the total PA tracker steps. This finding can be attributed to the lower proportion of steps-1 in the total steps composition among smartwatch users (ie, smartwatch users took fewer continuous regular walking steps [steps-1] in this study), as shown in Table 2. As the highest correlation between the smartphone and PA tracker step counts was observed for steps-1, we would not expect an increase in the correlation between the smartphone and the total PA tracker steps.

We applied mixed effects modeling to predict different step types (continuous regular walking, sporadic walking, and slow continuous walking) by using the smartphone step counts. Mixed effects models are a type of regression analysis and are especially useful in longitudinal studies with repeated measurements or when the measurements are made on cluster units [39]. Although we could fit mixed effects models with relatively high-performance metrics, the bootstrapping methods demonstrated wide prediction intervals. Therefore, estimating the daily number of steps by using the smartphone step counts without further precalibration would be imprecise and inaccurate. The best-fitted model was achieved for continuous regular walking (steps-1), which is consistent with the observation of the highest correlation between smartphone step counts and continuous regular walking (steps-1). On the basis of the models’ coefficients, we found that the postoperative period and a positive medical history were negatively associated with the total daily steps. The mixed effects models could also describe the variance in data between and within different individuals. We found that the variation between individuals in both the intercept and the slope of the PA tracker–smartphone steps was higher for sporadic walking (steps-2) and slow continuous walking (steps-3), which makes estimating these variables more difficult. In all 4 fitted models, the variance of the random effects intercept between individuals was more pronounced than that of the random effects slopes.

In this study, the PA tracker and the smartphone app obtained SUS score higher than the acceptable value, which was assumed to be 70 [21]. However, the SUS score cannot independently make absolute judgments about the *goodness* of a product. Factors such as success rate and the nature of the observed failures should play a prominent role in product usability [40]. During this study, we observed 1 smartphone app failure, which led to participant exclusion. This participant unintentionally removed the app from her smartphone and could not reinstall it because of technical issues. Furthermore, we found higher usability scores in patients aged <60 years and female patients. The effects of age and sex were analyzed in SUS applied for different products. A significant but not strong negative correlation has been demonstrated between SUS scores and age; however, no significant difference has been found in the mean SUS scores between female participants and male participants [21]. Some studies have also shown that the young adults and female participants were associated with higher PA tracker use [41,42].

### Strengths and Weaknesses of the Study

This longitudinal study is the first of its kind to evaluate the correlation between the daily steps recorded by a smartphone with the total number of steps in patients undergoing orthopedic surgeries for several weeks before and after surgery and in a nonpatient group. We also analyzed different walking types (regular continuous, sporadic, and slow continuous walking) and demonstrated that smartphones are more competent in capturing the steps during regular continuous walking. Detecting different gait patterns by smartphones and PA trackers has recently received considerable attention [43-45]. Indisputably, we must acknowledge the limitation that the validity of the 3 categories of steps measured by the PA tracker in this study has not yet been fully explored and must be scrutinized. Furthermore, our study had other limitations, such as the inability to obtain information regarding the smartphone use habits of the participants, including how and where the user carries the smartphone. Nevertheless, we used mixed effects modeling and random effects variables to account for individual differences to increase the generalizability of the findings. Another limitation of the study was that owing to the setting of the study, we could not use direct observation as the gold standard for counting the steps. However, to reduce data collection bias, we used a previously validated PA tracker that measured PA continuously 24/7.

### Implications and Future Research

In this study, we found a high correlation between the number of steps recorded by smartphones and the total number of daily steps. However, owing to the limitations and impact of participant dropouts and missing data, we recommend interpreting the findings with caution and conducting further investigations with larger sample sizes and more robust data collection methods. In addition, further investigations with larger sample sizes and more robust data collection methods are necessary to explore determining factors in the predictability of smartphone measurements and their role in remote patient monitoring. The study also demonstrated the predictive value of the postoperative period and positive medical history in estimating the total daily steps, but more homogenous samples may increase the precision of these prediction models. In future research, it would be valuable to compare the measurements of other well-known PA trackers with varying characteristics to smartphone measurements [46].

### Conclusions

This study highlights the potential of smartphones for monitoring changes in PA, showing a strong correlation between daily steps recorded by smartphones and total daily steps, especially during continuous walking. This finding suggests that smartphones could be a valuable tool for remote patient activity monitoring. However, accurately predicting the precise daily step counts from smartphone data still requires further investigation, as our results suggest that the current methods may lack the necessary precision and accuracy.

## Abbreviations

API: application programming interface
PA: physical activity
PROM: patient-reported outcome measure
REDCap: Research Electronic Data Capture
SUS: System Usability Scale
